# Heroin assessment in Spanish population-based studies: a scoping review

**DOI:** 10.1017/neu.2026.10081

**Published:** 2026-04-24

**Authors:** Ana Teijeiro, Ana García-González, Nerea Mourino, Sara Correia, Carla Guerra-Tort, Cristina Candal-Pedreira, Guadalupe García, Julia Rey-Brandariz, Mónica Pérez-Ríos

**Affiliations:** 1 Department of Preventive Medicine and Public Health, Universidade de Santiago de Compostela, Spain; 2 Preventive Medicine, Preventive Medicine Service, Complejo Hospitalario de A Coruña, Spain; 3 Epidemiology, Public Health and Health Services Evaluation, Instituto de Investigación Sanitaria de Santiago de Compostela, Spain; 4 CIBERESP, Spain; 5 University of A Coruña, University College of Nursing, Oza, A Coruña, Spain

**Keywords:** heroin, prevalence, Spain, scoping review, qualitative interview

## Abstract

**Introduction:**

Estimating the prevalence of use of substances such as heroin remains a challenge. The aim of this study is to identify the scientific publications in Spain that have used surveys to investigate heroin use, to describe their methodology and to contrast the formulation of the questions with users’ input on key aspects associated with use.

**Methods:**

A scoping review was conducted until November 2024 in MEDLINE (Ovid), EMBASE and Web of Science. The review included questionnaire-based research studies assessing heroin use in Spain. Information on study, population, data collection and consumption characteristics was compiled from each included study. In addition, in-depth interviews were conducted with Spanish heroin current users and ex-users.

**Results:**

Twenty-nine questionnaire-based research studies assessing heroin use in Spain were identified; none of them were specifically oriented to estimate and characterise heroin use at the population level. Most of the studies focused on specific population groups, mainly drug users, students, or inmates. The majority addressed lifetime, past-year, and past-month use, although users found the past 3 or 6 months more relevant. Few studies explored other use characteristics; however, interviews with heroin ex-users highlighted the importance of factors like route of administration and age of first use.

**Conclusions:**

The studies identified in this review vary in terms of target population, geographic scope, reference time frame, and data collection methods. Moreover, questionnaires rarely address additional characteristics of use that are considered relevant by former users. This review identifies areas for improvement to guide future studies and refine methodological approaches.

Significant outcomes

- No studies focuses on estimating and characterising Spanish heroin use.
- Studies focus on specific populations such as drug users, students and inmates.
- Characteristics of use are rarely asked about despite the importance given by users.

Limitations

- It is important to standardise surveys and incorporate users’ perspectives.
- Although all studies that asked about heroin use were included, none had heroin as a primary objective.
- The age of first use is a relatively underexplored factor, yet it is regarded as relevant by former users.

## Introduction

The abuse of illicit substances is one of the main public health problems in Western countries (Royo-Bordonada, 1997). Spain is among the European countries with the highest prevalence of drug use and the largest quantity of seised substances by weight, mainly heroin and cannabis, according to data from the European Union Drugs Agency (EUDA) (Vella *et al*., 2022).

Heroin is an opioid derived from morphine. It has different routes of administration and stimulates the reward system, generating a feeling of euphoria and reinforcing its consumption (National Institute on Drug Abuse, 2021). In the short term, its effects include drowsiness and a slowing of breathing and a decrease in heart function, and in cases of overdose, it may cause brain damage or even death. In the long term, it alters brain regions involved in decision making and behavioural control, promoting tolerance and physical dependence (San Juan Sanz, 2019; European Monitoring Centre for Drugs & Drug Addiction, 2021b). Heroin use is also associated with various comorbidities, such as infectious diseases, cardiovascular conditions, respiratory problems, and psychiatric disorders. In addition, it leads to social problems and social exclusion (European Monitoring Centre for Drugs & Drug Addiction, 2021a).

During the late 1970s, heroin consumption generated alarm amongst the general population in Spain due to its rapid expansion and its impact on youth mortality, both due to overdose and infection by the Human Immunodeficiency Virus (HIV) associated with intravenous consumption (Casanova Barbarà *et al.*, 2003). In light of this crisis, the need arose to develop surveillance systems to understand the epidemiological characteristics associated with heroin use and to design prevention and harm reduction strategies.

The most commonly used methodologies for assessing drug use include notification systems, ethnographic studies and population-based surveys (de la Fuente Hoz & Antó Boqué, 1991). The use of surveys allows for the systematic collection and comparison of data (Moya García, 2006). In Spain, the Government Delegation for the National Plan on Drugs has been carrying out two biennial surveys since the 1990s: the Survey on Alcohol and Other Drugs in Spain (EDADES) and the Survey on Drug Use in Secondary Education in Spain (ESTUDES). These surveys provide information to design and evaluate policies for the prevention of drug use and drug-related problems (Plan Nacional sobre Drogas, 2024; Plan Nacional sobre Drogas, 2025). However, estimating the prevalence of psychoactive substance use remains a challenge. Population-based surveys often use households as the sampling unit, which may exclude key groups of users. In addition, heroin use, commonly referred to as a ‘hard drug’, often has lower response rates in surveys due to the stigmatisation of its use, which makes it difficult to obtain accurate data (Organización de las Naciones Unidas, 2003).

The EDADES 2024 survey has estimated the prevalence of heroin use in Spain in the last year at 0.1%, reflecting a stabilisation of use at very low levels in recent years. However, despite the low reported prevalence, heroin use continues to have a significant social and health impact. Among all drugs, legal and illegal, heroin generates the greatest damage to health, in addition to requiring a significant proportion of health resources due to hospital emergencies and treatment for dependence (Vella *et al*., 2022). Therefore, the aim of this study is to identify the scientific publications in Spain that have used surveys to investigate heroin use, describe their methodology and contrast the formulation of the questions with the perception of users and ex-users on key aspects associated with use.

## Methods

A scoping review was conducted in accordance with the PRISMA-ScR 2018 (Preferred Reporting Items for Scoping Reviews) guidelines (Page *et al*., 2021).

### Search strategy

The search included records published up to November 2024 in MEDLINE (Ovid), EMBASE and Web of Science (WoS), after applying a search strategy pre-designed by three experts (ATT, AGG, and NM). Free terms and MeSH terms such as ‘heroin’, ‘prevalence’ and ‘Spain’ were included in the search strategy. The complete search strategy can be found in Table 1 of the Supplementary Material. No restrictions on publication date, study period, study design, sample size, or language were applied in the search.

A manual review of the references of the included studies was performed to ensure the inclusion of all possible studies not obtained in the initial search.

### Inclusion and exclusion criteria

The review included questionnaire-based research studies assessing heroin use in Spain. We took into account studies carried out in any population, with any source of recruitment and time frame to which consumption was evoked, regardless of whether the main objective of the study was to estimate the prevalence of heroin use.

Studies with the following characteristics were excluded: those whose study population consisted exclusively of previously identified heroin users and those in which the question focused on drugs in general. In addition, studies that assessed heroin use disorder as a clinical diagnosis (either through the clinical history or through validated scales), as well as those that assessed intoxications in hospital emergency departments or drug-related police arrests, were excluded. Reviews, conference abstracts, letters to the editor, editorials, opinion articles, preprints, protocols and retracted articles were also excluded.

### Selection of articles and synthesis of evidence

After eliminating duplicate articles, seven investigators independently reviewed and assessed the eligibility of the titles and abstracts of all articles obtained in the search. Potentially relevant articles were read in full text to ensure that they met the inclusion criteria. Any disagreement on the inclusion or exclusion of an article was resolved by consensus among the disagreeing investigators.

Extraction of information from the selected articles was performed using an extraction table designed ad hoc in an Excel spreadsheet. The information was extracted manually by three investigators (ATT, AGG, and NM). Discrepancies were discussed and resolved by consensus.

For each included study, the following information was extracted:

- Characteristics of the study: 1) first author; 2) year of publication; 3) year or period studied; 4) geographic scope: international, national, regional, or local; 5) autonomous community where the study was conducted (when applicable); 6) study design; 7) name of the study or survey from which the data were obtained (when applicable); and 8) main objective of the study: to assess heroin use exclusively, to assess psychoactive drug use including heroin, to assess drug use in relation to mental health disorders, to assess risk practices, or other objectives.
- Characteristics of the population under study: 1) source of recruitment: health or social-health care setting, educational setting, penitentiaries, distribution and consumption settings, respondents’ homes (selected through census/official census), and internet (social networks and websites); 2) population group: general population, students (high school and university), mothers, inmates, people with HIV, people with mental disorders, people in unhoused situations, and drug users; 3) age in years: range and/or mean age; 4) sample size (<500, 500–1000, >1000, unspecified); 5) convenience sampling (yes/no); 6) gender of participants (male, female, or both); and 7) offering incentives to participants (yes/no).
- Data collection characteristics: 1) method of questionnaire administration: self-administered, telephone, personal interview by trained or untrained interviewer, or unspecified; 2) validation of heroin use with biomarkers (yes/no); 3) literals; 4) time frame to which use is evoked: lifetime, last year, last 6 months, last 3 months, last month, last week, current, other, unspecified; 5) frequency of heroin use; and 6) characteristics related to use: route of administration or age at first use, among others.
- Characteristics of use: prevalence of heroin use as a percentage (%), categorised by study population, regardless of the time frame in which use was evoked.

### Interviews with key informants

Non-profit organisations, foundations, and drug-dependence care units in Galicia (Spain) were contacted with the aim of identifying current or former heroin users. In-depth face-to-face interviews were conducted with volunteers. Inclusion criteria required participants to be current or former users seeking treatment, while people with violent tendencies or who were uncooperative were excluded. Although an attempt was made to ensure the participation of different genders, only males participated. Based on the questions identified in the previous review, semi-structured questionnaires were designed to be completed by current and former users to assess the relevance and clarity of the questions included in previous studies and to identify any missing domains that should be incorporated. In addition, they were asked about the importance of various questions identified in the reviewed literature concerning first use, types and routes of heroin administration, use by family and friends, and other related topics.

The script used by the interviewer (member of the research team) for the open-ended questions is shown in Table 2 of the Supplementary Material. The audio of all interviews was recorded with the prior consent of the participants. The information obtained from the interviews was thematically analysed to identify and compare the informants’ opinions.

All participants signed informed consent after being duly informed by the interviewer about the objectives of the study. The Ethics Committee of Santiago de Compostela considered that evaluation of this study was not necessary, since no personal participant data was used.

## Results

**Figure 1.**
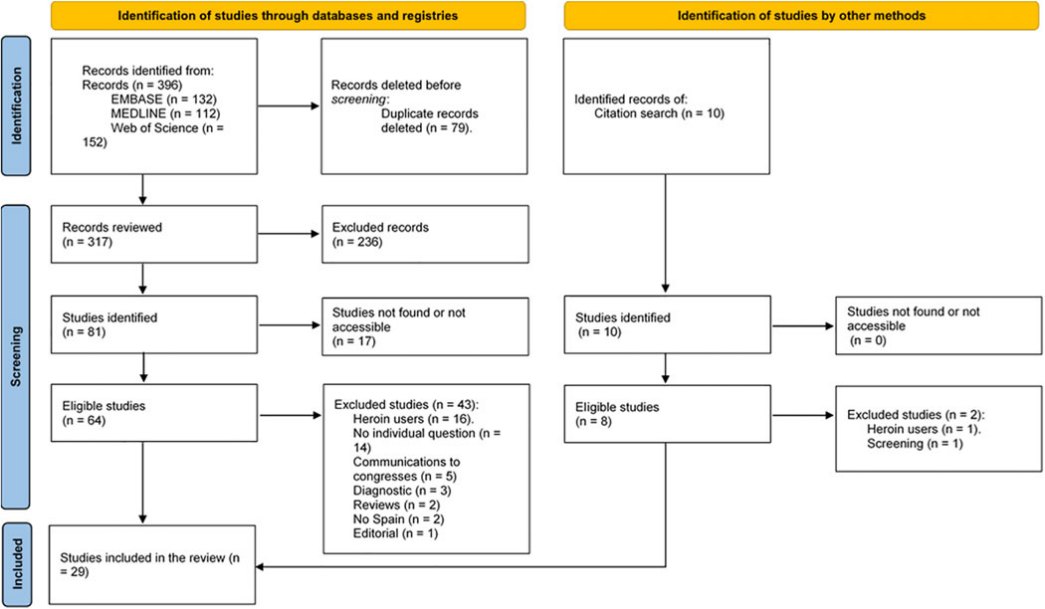
Figure 1 long description. Flowchart of study selection.

Figure 1 shows the flowchart of the study selection. A total of 396 records were identified through database searches. After screening titles and abstracts, 236 records were excluded, and 17 studies could not be retrieved. Of the 64 studies assessed for eligibility, 43 were excluded for the following reasons: population consisted exclusively of previously identified heroin users (*n* = 16), studies in which the research question focused on drugs in general rather than specifically on heroin (*n* = 14), conference abstracts (*n* = 5), studies assessing heroin use disorder as a clinical diagnosis (*n* = 3), reviews (*n* = 2), editorials (*n* = 1), and studies not conducted in Spain (*n* = 2). Therefore, following this initial screening, 21 studies were preselected for inclusion. In addition, 10 further records were identified through manual review of the references of the selected studies; of these, 2 were excluded (one during initial screening for being a review and another due to the exclusive inclusion of previously identified heroin users). Finally, 29 studies met the inclusion criteria and were included in the review (Bravo Portela *et al*., 1996; Royo-Bordonada, 1997; Moncada Ribera & Perez Gonzalez, 1998; Mendoza Berjano *et al*., 1998; Perez Gonzalez *et al*., 1999; Bravo Portela *et al*., 2000; Suelves *et al*., 2003; Bravo *et al*., 2004; Prinzleve *et al*., 2004; Busquets *et al*., 2005; Folch *et al*., 2006; Garcia-Algar *et al*., 2009; Vazquez, 2010; Etcheverry *et al*., 2011; Caravaca-Sánchez & Wolff, 2016; Maremmani *et al*., 2016; Caravaca-Sánchez *et al*., 2018; Font-Mayolas *et al*., 2019; Fuster-RuizdeApodaca *et al*., 2019;Vazquez *et al*., 2019; Caravaca-Sanchez & Garcia-Jarillo, 2020; Caravaca-Sánchez & Wolff, 2020; Guillen *et al*., 2020; Caravaca-Sánchez *et al*., 2021; Whitlock *et al*., 2021; Parro-Torres *et al*., 2022; Incera-Fernandez *et al*., 2022; Garcia-Perez *et al*., 2023; Perez *et al*., 2023).

### Characteristics of the included studies

The studies were published between 1996 and 2023. More than half of the studies (15/29) were published within the last 10 years (Figure 1 of the Supplementary Material). Most of the studies (27/29) had a cross-sectional design; the remaining studies had a longitudinal (1/29) or cohort (1/29) design. No study aimed to assess heroin use exclusively. Most (13/29) focused on assessing psychoactive drug use in general, among these, heroin (Table 1 and Supplementary Table 3).

**Table 1.** Main characteristics of the included studies according to recruitment source

	Health or social-health care (*n* = 11)	Educational (*n* = 6)	Penitentiaries (*n* = 5)	Distribution and consumption (*n* = 4)	Respondents’ homes (*n* = 2)	Internet (*n* = 1)	Total (*n* = 29)
Main objective of the study							
Heroin use	0	0	0	0	0	0	0
Psychoactive substances use	7	5	1	0	1	1	15
Substance use and mental health disorders	2	1	2	0	0	0	5
Risky practices	0	0	1	4	0	0	5
Others^1^	2	0	1	0	1	0	4
Population groups							
General population	0	0	0	0	2	0	2
Drug users	4	0	0	4	0		19
Students	0	6	0	0	0	0	6
People with HIV	3	0	0	0	0	0	3
Inmates	0	0	5	0	0	0	5
Others^2^	4	0	0	0	0	0	4
Sample size							
<500	7	0	3	2	1	0	13
500–1000	0	2	1	0	1	0	4
>1000	3	4	1	2	0	1	11
Not specified	1	0	0	0	0	0	1
Questionnaire administration method							
Personal interview	8*	1	0	4	2	0	15
Telephone	0	0	0	0	0	0	0
Self-administered	3	5	5	0	0	1	14
Not specified	1*	0	0	0	0	0	1
Validation with biomarkers**							
Yes	2	0	0	0	0	0	1
No	9	6	5	4	2	1	27
Time frame***							
Lifetime	3	5	0	0	1	0	9
Last year	4	2	0	0	0	0	8
Last 6 months	0	2	2	1	0	0	5
Last 3 months	0	0	3	0	0	0	5
Last month	5	4	0	3	1	0	13
Current	1	0	0	0	0	0	1
Other^3^	2	0	0	0	0	1	2
Not specified	1	0	0	0	0	0	1
Other characteristics of heroin use***							
Route of administration	3	0	0	2	0	0	5
Age of first use	1	3	0	1	0	0	5
Other^4^	2	2	0	1	1	1	7

1 1) to assess compliance with drug use-related objectives in a Health Plan; 2) to explore the prevalence of sexual victimisation within prison and the association of sexual violence and substance use experience, 3) to examine the vulnerability of homeless women, and 4) to determine the influence of perceived social support and resilience on alcohol and drug use in imprisoned women.

2 Mothers, people with mental problems, people in unhoused situations.

3 During pregnancy, last 2 years, last 2 weeks.

4 Perception of drug availability, family substance use, perceived health risks, use habits, use patterns, treatment received, social factors related to use, quantity consumed.

*One study collected information using two methods: personal interview and another method not specified.

**The number refers to the studies that used biomarkers.

***Many studies asked for more than one time frame and several characteristics of use.

With regard to geographic scope, 16 studies focused on local areas, seven were regional, four were national, and two were international (Supplementary Table 3). In those studies that specified the autonomous community in which the study was conducted, the communities with the greatest number of studies were Catalonia (11/29) and the Community of Madrid (9/29). It is noteworthy that, in autonomous communities with more than one study, the majority focused on drug users, except in the Region of Murcia, where four of the five studies focused on inmates (Table 2).

**Table 2.** Number of studies carried out by autonomous community according to the study population (*n* = 23)

Autonomous community	Population	Total
*Andalusia*	Drug users (*n* = 2)	4
	People with HIV (*n* = 1)	
	Inmates (*n* = 1)	
*Aragon*	People with HIV (*n* = 1)	1
*Principality of Asturias*	People with HIV (*n* = 1)	1
*Balearic Islands*	People with HIV (*n* = 1)	1
*Cantabria*	People with HIV (*n* = 1)	1
*Castile-La Macha*	Inmates (*n* = 1)	1
*Castile and Leon*	People with HIV (*n* = 1)	1
*Catalonia*	Drug users (*n* = 5)	11
	Students (*n* = 2)	
	People with HIV (*n* = 2)	
	General population (*n* = 1)	
	Mothers (*n* = 1)	
*Galicia*	Drug users (*n* = 1)	3
	People with HIV (*n* = 1)	
	Students (*n* = 1)	
*Community of Madrid*	Drug users (*n* = 3)	9
	People with HIV (*n* = 3)	
	People in unhoused situations (*n* = 2)	
	Students (*n* = 1)	
*Region of Murcia*	Inmates (*n* = 4)	5
	People with HIV (*n* = 1)	
*Basque country*	People with HIV (*n* = 1)	1
*Valencian community*	Drug users (*n* = 2)	4
	People with HIV (*n* = 1)	
	Inmates (*n* = 1)	

### Characteristics of the populations studied

The sample sizes of the studies ranged from 30 to 36 984, with the majority being less than 1,000 participants (17/29). The most studied populations were drug users (9/29), followed by students (6/29) and inmates (5/29). The age of individuals studied ranged from 11 to 78 years. Most studies included both men and women, except for five studies that included only women and two that included only men. Participants were recruited primarily from health care facilities (11/29), educational facilities (6/29), penitentiaries (5/29), and drug use and distribution settings (4/29) (Table 1). Three studies offered incentives to their participants (Supplementary Table 3).

### Data collection characteristics

Approximately half of the studies collected information through face-to-face interviews (15/29) and the other half with self-administered questionnaires (14/29) (Table 1). In one third of the studies, a trained interviewer was responsible for data collection (five of the 15 interviews and four of the 14 questionnaires). It is noteworthy that seven of the nine studies in drug users were conducted face-to-face. Nearly 90% of the studies (26/29) used ad hoc questionnaires, while the remaining three focused on different target populations (people living with HIV, the general population, and students), thereby limiting the comparability of the questions included. Two studies validated the questionnaires with urinalysis and meconium analysis (Supplementary Table 3).

The most frequent time frames to which use was evoked were last month (13/29), lifetime (9/29) and last year (8/29). In addition, five studies also asked for the route of administration and another five for the age at first use (Table 1). Six studies included the verbatim questions used to ask about heroin use (Table 3).

**Table 3.** Verbatim questions in the studies that included them (*n* = 6)

First author, year of publication	Literals
Garcia-Perez A, 2023	‘Have you ever used heroin?’ Response options: Yes/No
Incera-Fernandez D, 2022	‘If, during the last 24 months, they had used drugs or substances before or during a sexual encounter to facilitate, intensify, or prolong sexual activity’ Possible answers: Yes/No for each of the drugs listed in the questionnaire.
Caravaca-Sánchez F, 2020	‘How often in the past 3 months they had used five types of substances—alcohol, cannabis, cocaine, heroin, and psychotropic medications without a prescription’ Response options: scale from 1 to 5, where 1 is never and 5 is every day.
Font-Mayolas S, 2019	Recent use (last month) of 9 drugs (tobacco, alcohol, cannabis, cocaine, heroin, volatile inhalants, speed/amphetamines, hallucinogens and Spice). Response options: scale from 1 to 3, where 1 is never, 2 is once a month and 3 is two or more times a month.
Caravaca-Sánchez F, 2018	‘Have you consumed any [or several] of these substances (alcohol, cannabis, cocaine, heroin, or crack) during the 6 months prior to your current prison sentence?’ ‘Have you consumed one [or more than one] of the following substances (alcohol, cannabis, cocaine, heroin, or crack) during the last 6 months, while in prison?’ Response options: Yes/No
Caravaca-Sánchez F, 2016	‘Have you consumed any or several of the following substance (alcohol, cannabis, cocaine, heroin, crack cocaine, psychotropic [without medical prescription], and ecstasy) during the previous 6 months in prison?’ Response options: Yes/No.

### Consumption characteristics

Prevalences of heroin use varied widely in all population groups from 0.1% to 85.0%. The highest prevalences were estimated in drug users (85.0%), followed by prisoners (26.9%) and homeless people (24.9%). In general, the lowest prevalences were obtained in studies in the general population and in students, except for a study among drug users in which only 0.1% used heroin during sexual practice (Table 4).

**Table 4.** Prevalence of use among different population groups, regardless of the time frame

Population	Value/range of prevalence of heroin use (%)
General population	0.2–1.7
Drug users	0.1–85.0
Students	0.2–1.0
People with HIV	1.3–8.0
Inmates	2.4–26.9
People in unhoused situations	4.7–24.9
People with mental problems	3.0
Mothers	0.3 (survey), 4.7 (meconium analysis)

### Interviews with key informants

We conducted three interviews with heroin former users (February 24, 2025). Five themes were identified: specific stages of consumption; types, routes, and amount consumed; environment; relapses; and frequency and time frame of the consumption.

All informants agreed that it was important to ask about past heroin use and that users or ex-users easily remember the age of first use, periods of use associated with positive or negative life experiences, and use in life stages such as pregnancy or adolescence. In addition, there was consensus on the importance of inquiring about the route of administration and how it has changed over time, as well as about heroin use in user’s environment and any episodes of relapse.

*‘It makes a difference how you administer.’ (‘Es importante la forma de administrar.’) (IC3)*

*‘If your partner is using, I think it’s almost impossible to quit, if you both use.’ (‘Si tu pareja consume, yo creo que es casi imposible dejarlo, si los dos consumen.’) (IC1)*

*‘Because I am a human being, my whole environment affects me.’ (‘Yo como individuo que soy, todo mi entorno, me afecta.’) (IC3)*

*‘Relapses are important, for me it is the most important [question].’ (‘Las recaídas son importantes, para mí lo es todo.’) (IC1)*

Two of the informants pointed out that it is important to ask about the amount of heroin consumed and that they would be able to indicate how much heroin they consume or used to consume. However, both considered that it is not important to ask about the frequency of use. The other informant pointed out that frequency should be addressed, noting that during periods of use, consumption tends to be daily. As for the time frame, two respondents felt that questions should focus on the past 3 months, while the other recommended referring to the past 6 months.

*‘Yes, the amount, how many times a day, if it was daily, or not.’ (‘Sí, la cantidad, durante cuántas veces al día, si era diario, si no.’) (IC2)*

*‘But it’s just that it’s hard, daily work [quitting drugs]. I don’t think it can be summed up in one month.’ (‘Pero es que es un trabajo duro y diario. Yo creo que no se puede resumir en un mes.’) (IC1)*

*‘Yes, for a minimum three months.’ (‘Sí, para el mínimo tres meses.’) (IC1)*

*‘I would put six months as well, minimum.’ (‘Pondría los seis meses también, mínimo.’) (IC3)*

## Discussion

No study has been published in Spain aimed at estimating and characterising heroin use at the population level. Most of the studies carried out include men and women, focus on specific population groups and assess drug use in general. The autonomous communities in which the largest number of studies are concentrated are Catalonia and the Community of Madrid. Most of the studies have small sample sizes and have been conducted on drug users, students, or inmates. Most of the studies on drug users obtained information through face-to-face interviews. They mainly addressed lifetime, past year and past month use. Few studies asked about the route of administration and age at first use.

Based on data from the EDADES 2024 survey, it is estimated that, in Spain, the prevalence of heroin use in the past year in the general Spanish population has always been less than 0.5% since the survey began three decades ago (Plan Nacional sobre Drogas, 2024). It is possible that this low prevalence is one of the reasons why specific population-level studies focusing on heroin use are not carried out and why they are analysed in association with other psychoactive substances. This approach is partly justified by the fact that heroin users are usually poly-drug users (Salani *et al*., 2016), but it precludes detailed knowledge of the epidemiology and topography associated with heroin use.

The approach of the published research studies addressing heroin use focuses on previously identifying groups in which the prevalence of use is higher, such as users of other drugs, prison inmates and people in unhoused situations. The first two groups correspond to the populations that are most frequently studied in Spain (Table 4). On the other hand, people in unhoused situations are scarcely represented in the studies, despite being one of the populations with the highest prevalence of drug use (Doran *et al*., 2018); this underrepresentation is particularly concerning given the close link between homelessness and drug use (Hungaro *et al*., 2020). Researchers have noted that, despite the higher rate of heroin use in these populations, the limited accessibility to this demographic restricts the study sample sizes.

As for gender, this is not assessed in the studies identified, and an approximation is made based on sex. Most of the studies include both men and women, although there are two specific studies on men and five on women (Salani *et al*., 2016). Despite heroin use is predominantly male, it is essential to examine substance use in both sexes, since conditioning factors and consumption patterns differ (Vigna-Taglianti *et al*., 2016). Among the five studies on women, three were conducted in prison settings, where drug abuse is the main reason for incarceration and a major health concern (Henderson, 1998), highlighting the importance of studying this population.

Notably, only men agreed to participate in the qualitative interviews included in this review, which may have led to a gender bias. Previous studies on Spanish female drug users have reflected the persistent underrepresentation of women in substance use research and treatment programmes, often attributed to stigma, fear of disclosure, distrust toward professionals, and a lack of support from their immediate social environment (Shirley-Beavan *et al*., 2020; Aguila-Morales & Clua-Garcia, 2024). Another factor that may have influenced non-participation is the participants’ prior lack of knowledge of the sex of the interviewer, as evidence suggests that women who use drugs frequently report feeling more comfortable and more inclined to disclose personal information when interacting with female professionals (Aguila-Morales & Clua-Garcia, 2024). Future research should adopt gender-sensitive recruitment strategies and account for these factors to ensure equitable representation.

Heroin use, in addition to causing overdose, is associated with various pathologies (European Monitoring Centre for Drugs & Drug Addiction, 2021a, European Monitoring Centre for Drugs & Drug Addiction, 2021b), which means that it is one of the drugs that causes the most emergency room visits (Vella *et al*., 2022). This could explain why most studies recruit participants in health care settings. However, this method of recruitment may introduce a selection bias, as a proportion of people who use heroin do not seek care when they suffer adverse health effects (Rogeberg & Pedersen, 2021), or they seek attention that goes unnoticed (Dunn *et al*., 2011).

Studies on drug use are highly susceptible to social desirability bias, particularly when participants are required to self-report stigmatised behaviours such as heroin use (Kaushal, 2014). Interviewer-administered surveys on heroin use may lead respondents to conceal this socially stigmatised behaviour, resulting in underreported data. Respondents may avoid giving honest answers due to fear of judgement or social pressure (Tourangeau & Yan, 2007). In contrast, computer-assisted self-interviewing (CASI) and audio-CASI (ACASI) have demonstrated greater effectiveness in eliciting accurate responses by reducing perceived social presence and the threat of judgement or repercussions (Tourangeau & Yan, 2007). Given that most data on heroin use in Spain are derived from face-to-face interviews, it is plausible that consumption estimates are influenced by social desirability bias, although the magnitude of this effect cannot be determined without mode-comparison studies or external validation data (e.g., biomarkers). In fact, the former users interviewed agreed that consumption is also concealed in questionnaires. Notably, two of the included studies combined face-to-face questionnaires with biological assessments (meconium and urine analyses), one of which revealed use underreporting (Tourangeau & Yan, 2007; Garcia-Algar *et al*., 2009; Kaushal, 2014). Face-to-face or self-administered questionnaires should be supported by strategies that enhance disclosure accuracy, including introductory statements that present risk behaviours in a non-judgemental context, pilot testing to assess item sensitivity, indirect questioning techniques (such as the randomised response and item count methods), and, where feasible, biomarker validation to objectively corroborate self-reported data (Tourangeau & Yan, 2007; Latkin *et al*., 2016; Mourino *et al*., 2021). Further research is needed to quantify the extent of misreporting and to keep identifying effective methods for minimising bias in studies involving stigmatised substance use.

In addition, the perceptions of which aspects matter in developing drug-use questionnaires may have been also influenced by the fact that interviews were conducted exclusively with assistance-seeking heroin former users. Such participants may differ from active or non–treatment-seeking users in sociodemographic and personality characteristics, disorder severity, patterns of use, and motivation for cessation (Ray *et al*., 2017). Given the potential recruitment bias, the findings may not be generalisable to all heroin users. Despite recruitment challenges, future research should seek to recruit participants across diverse stages of drug use and rehabilitation to ensure a more comprenhensive understanding of their experiences.

Population-based surveys with large sample sizes and assessments covering multiple substances are primarily designed to estimate prevalence. As a result, they often omit or only superficially capture key dimensions of drug use (European Union Drugs Agency, 2009). Consistent with this limitation, the studies included in this review rarely captured certain characteristics of heroin use that former users from qualitative interviews considered particularly important, such as quantity consumed, intensity of use, route of administration, age at first use, and negative peer influence. This limited measurement under-represents important characteristics of drug users and restricts both the development of more comprehensive future questionnaires and the interpretability and generalisability of findings.

Coinciding with the timeframes established in EDADES (Plan Nacional sobre Drogas, 2024) and ESTUDES (Plan Nacional sobre Drogas, 2025) to evoke memory of consumption, most of the studies refer to the last month, last year and once in a lifetime. Some studies adopted intermediate recall periods, aligning with recommendations from former heroin users participating in the qualitative interviews, who emphasised that very short or overly broad timeframes fail to capture typical patterns of use and relapse; thus, extending recall beyond a single month is essential for improving accuracy and distinguishing current from former users. Recent evidence supports the validity of three- to six-month periods, which balance accuracy and feasibility while minimising underreporting due to relapse and recall bias (Tang *et al*., 2024). Former heroin users also consider that questionnaires should capture quantity consumed alongside frequency and usage patterns (e.g., daily), as these combined dimensions provide a more accurate depiction of typical consumption dynamics. All these variables should be incorporated into survey design to enhance the reliability of prevalence estimates and strengthen the utility of findings for evidence-based public health interventions.

Former users from the qualitative interviews emphasised the importance of capturing the route of administration, noting that initiation typically occurs via one route followed by transition to another, often toward routes associated with greater harm. The route of administration shows substantial variation at both individual and population level, and it is associated with distinct adverse health effects; for example, increased intravenous use may hinder HIV control efforts and lead to greater severity of dependence compared with other routes of administration (Gossop *et al*., 2004). Evidence indicates that individuals who initiate heroin use via injection progress to daily use more rapidly than those who begin by inhalation or snorting, highlighting pharmacokinetic differences that may affect the optimal timing of interventions (Hines *et al*., 2017). Of the five studies that asked about the route of administration, only one included the options considered (smoked, injected, snorted, oral, inhaled and intrarectal) (Fuster-RuizdeApodaca *et al*., 2019). Accordingly, surveys should systematically collect information on lifetime and current routes of administration, assess users’ awareness of route-specific risks, and document complications previously experienced.

Capturing the context of consumption is also important, as many users administer drugs via parenteral routes in public or improvised settings (e.g., streets, cars, parks). These behaviours not only reflect high-risk practices but also expose the broader population to hazards such as accidental needle-stick injuries from discarded syringes and increased visibility of drug-related activities (Moore, 2018; Valencia *et al*., 2021). Identifying the locations where drug use predominantly occurs, in both urban and rural contexts, can improve risk stratification and enable more precise targeting of harm-reduction and treatment interventions, reaching both individuals actively seeking assistance and other users who have not yet engaged with services. Focusing interventions in areas of frequent consumption can facilitate the establishment of strategically located supervised consumption spaces, the promotion of syringe service programmes and access to naloxone, and the provision of opioid agonist therapy and screening for HIV, tuberculosis, and hepatitis B and C; such targeted interventions would improve public health outcomes while reducing healthcare costs associated with hospital admissions or treatment due to drug overdoses, injection-related infections, chronic diseases, and accidental needle-stick injuries among non-users (Wenger *et al*., 2011; Moore, 2018; O’Rourke *et al*., 2019; Valencia *et al*., 2021).

With regard to the age of first heroin use, former users remembered it easily, reporting first consumption between 16 and 23 years of age. U.S. studies reported a mean initiation age of 17.7 years in 2017, reflecting a gradual increase since 2004 (Alcover & Thompson, 2020). Similarly, data from the latest EDADES report from 2024 show that, in Spain, age of initiation has increased since 1999, with a current mean of 21.6 years among the general population (Plan Nacional sobre Drogas, 2024). Among students, according to the ESTUDES report from 2025, initiation has remained stable (Plan Nacional sobre Drogas, 2025). During adolescence, substance use initiation is often influenced by peer groups and the fear of social exclusion. Qualitative interviews with former heroin users reflect that partner influence reinforces ongoing consumption and makes cessation particularly difficult. These findings underscore the importance of including age of initiation in epidemiological surveys and highlight the need to consider social and familial contexts when designing early, comprehensive health education initiatives.

For the correct interpretation of the results obtained, it should be taken into account that this study has limitations. We did not have access to the full text of 17 of the studies identified in the search, which may have resulted in the loss of potentially relevant information, although from the information in their abstracts, they did not appear to meet the criteria. Although all studies that asked about heroin use were included, none had heroin as a primary objective. The verbatim questions used to assess consumption were included in only six studies, so it is not possible to guarantee that the information obtained in the different studies is comparable. Another limitation of this study is that only former users of heroin were included. Therefore, there may be differences between their opinions and those of users who are not seeking assistance (Ray *et al*., 2017). Finally, this review includes only studies conducted in Spain, and its applicability to other countries may be limited.

Despite its limitations, this study also has some strengths; thus, this review provides a preliminary overview of how heroin use is assessed in questionnaire-based studies conducted in Spain. To our knowledge, this is the first study to address this topic while incorporating the perspectives of former heroin users on the most relevant aspects to consider when collecting data on consumption. Although it reveals gaps and inconsistencies in current survey practices, the findings offer a foundation for future methodological improvements. The methodological contribution of this study is significant, as there is a lack of prior reviews specifically addressing how heroin use is assessed at the population level. This analysis represents a crucial preliminary step for population-based surveys aimed at estimating the prevalence of heroin use, since designing a questionnaire without reference to existing validated instruments, as is the case in most of the studies included, should be avoided. Therefore, conducting a review that compiles available questions and evaluates their strengths and weaknesses is essential to support the development of more robust survey instruments. When combined with insights from people who use heroin, this approach facilitates data interpretation and informs potential improvements. Although this study focuses on a single country, which may a priori limit the generalisability of its findings, it provides valuable methodological guidance with relevance beyond Spain, given that heroin use shares a broadly similar pattern across developed countries.

In conclusion, the studies identified in this review vary in terms of target population, geographic scope, reference time frame, and data collection methods. Moreover, questionnaires rarely address key aspects for users such as characteristics of use, age at first use, peer influence or route of administration, and none of the studies assessed relapse, despite its relevance as highlighted by former heroin users. Including verbatim survey questions in subsequent studies and incorporating the perspectives of both male and female users, as well as current and former users who are not actively seeking assistance, would further enhance the interpretation and comparability of findings. Given these limitations, improving survey standardisation and data consistency is essential. While this review identifies areas for improvement, its findings should be interpreted with caution and used to guide future studies and refine methodological approaches.

## Supporting information

10.1017/neu.2026.10081.sm001 Teijeiro et al. supplementary material Teijeiro et al. supplementary material

## Supplementary material

The supplementary material for this article can be found at https://doi.org/10.1017/neu.2026.10081.

## Figure 1 Long description

The flowchart details the process of selecting studies for a review. It is divided into two main sections: Identification of studies through databases and registries, and Identification of studies by other methods. In the first section, records are identified from various databases: EMBASE, MEDLINE, and Web of Science. Duplicate records are deleted before screening. The remaining records are reviewed, and excluded records are noted. Studies not found or not accessible are also indicated. Eligible studies are then identified, and further exclusions are made based on specific criteria such as heroin users, no individual question, communications to congresses, diagnostic studies, reviews, studies not in Spain, and editorials. The second section involves identifying records from a citation search. Studies not found or not accessible are noted, and eligible studies are identified. Further exclusions are made based on criteria such as heroin users and screening. The final number of studies included in the review is shown.

Navigate back to Figure 1.
