# Supplementary material for: Heroin assessment in Spanish population-based studies: a scoping review
Source: Acta Neuropsychiatr. 2026 Apr 24;38:e43. doi: 10.1017/neu.2026.10081 (PMC13280516; doi:10.1017/neu.2026.10081)
Supplement: Teijeiro et al. supplementary material [file S0924270826100817sup001.docx]

**SUPPLEMENTARY MATERIAL**

Supplementary Table 1. Search strategy.

| Embase **<1974 to 2024 November 13>**  Ovid MEDLINE(R) ALL **<1946 to November 13, 2024>** | | |
| --- | --- | --- |
| 1 | Heroin/ or diamorphine/ or heroin.ab,ti. | 53.542 |
| 2 | Spain/ | 209.326 |
| 3 | (spain or espagne or espana or spagna or spain or espagne or espana or spanien or spagna or catalunya or catalonia or catalogne or cataluna or catala or barcelon* or tarragona or lleida or lerida or girona or gerona or valencia* or castello* or alacant or alicant* or murcia* or (cartagen* not indias) or andalu* or sevill* or granad* or huelva or almeria or cadiz or jaen or malaga or (cordoba not argentin*) or extremadura or caceres or badajoz or madrid or castilla or salamanca or zamora or valladolid or segovia or soria or palencia or avila or burgos or (leon not (france or clermont or rennes or lyon or USA or mexic*)) or galicia or gallego or compostela or vigo or corun* or ferrol or orense or ourense or pontevedra or lugo or oviedo or gijon or asturia* or cantabr* or santander or vasco or euskadi or basque or bilbao or bilbo or donosti* or "san sebastian" or vizcaya or bizkaia or guipuzcoa or gipuzkoa or alava or araba or vitoria or gasteiz or navarr* or nafarroa or pamplona or iruna or irunea or logron* or rioj* or aragon* or zaragoza or teruel or huesca or mancha or "ciudad real" or albacete or cuenca or (toledo not (ohio or us or usa or OH)) or (guadalajara not mexic*) or balear* or mallorca or menorca or ibiza or eivissa or palmas or lanzarote or canari* or tenerif* or ceuta or melilla).cp,ti. | 720.190 |
| 4 | 2 or 3 | 818.719 |
| 5 | prevalence/ or ("Prevalence" or "Epidemiology" or "cross-sectional" or "population-based" or survey).ab,ti. | 5.310.634 |
| 6 | epidemiology/ | 297.188 |
| 7 | 5 or 6 | 5.497.877 |
| 8 | 1 and 4 and 7 | 329 |
| 9 | remove duplicates from 8 | 244 |
| Web of Science Core Collection **<Thu Nov 14 2024>** | | |
| 1 | (TI=(diamorphine or heroin)) OR TS=(diamorphine or heroin) Editions: WOS.SCI,WOS.SSCI | 24.038 |
| 2 | (TS=("Prevalence" or "Epidemiology" or "cross-sectional" or "population-based" or survey)) OR TI=("Prevalence" or "Epidemiology" or "cross-sectional" or "population-based" or survey) Editions: WOS.SCI,WOS.SSCI | 3.240.039 |
| 3 | (TS=(spain or espagne or espana or spagna or spain or espagne or espana or spanien or spagna or catalunya or catalonia or catalogne or cataluna or catala or barcelon* or tarragona or lleida or lerida or girona or gerona or valencia* or castello* or alacant or alicant* or murcia* or (cartagen* not indias) or andalu* or sevill* or granad* or huelva or almeria or cadiz or jaen or malaga or (cordoba not argentin*) or extremadura or caceres or badajoz or madrid or castilla or salamanca or zamora or valladolid or segovia or soria or palencia or avila or burgos or (leon not (france or clermont or rennes or lyon or USA or mexic*)) or galicia or gallego or compostela or vigo or corun* or ferrol or orense or ourense or pontevedra or lugo or oviedo or gijon or asturia* or cantabr* or santander or vasco or euskadi or basque or bilbao or bilbo or donosti* or "san sebastian" or vizcaya or bizkaia or guipuzcoa or gipuzkoa or alava or araba or vitoria or gasteiz or navarr* or nafarroa or pamplona or iruna or irunea or logron* or rioj* or aragon* or zaragoza or teruel or huesca or mancha or "ciudad real" or albacete or cuenca or (toledo not (ohio or us or usa or OH)) or (guadalajara not mexic*) or balear* or mallorca or menorca or ibiza or eivissa or palmas or lanzarote or canari* or tenerif* or ceuta or melilla)) OR TI=(spain or espagne or espana or spagna or spain or espagne or espana or spanien or spagna or catalunya or catalonia or catalogne or cataluna or catala or barcelon* or tarragona or lleida or lerida or girona or gerona or valencia* or castello* or alacant or alicant* or murcia* or (cartagen* not indias) or andalu* or sevill* or granad* or huelva or almeria or cadiz or jaen or malaga or (cordoba not argentin*) or extremadura or caceres or badajoz or madrid or castilla or salamanca or zamora or valladolid or segovia or soria or palencia or avila or burgos or (leon not (france or clermont or rennes or lyon or USA or mexic*)) or galicia or gallego or compostela or vigo or corun* or ferrol or orense or ourense or pontevedra or lugo or oviedo or gijon or asturia* or cantabr* or santander or vasco or euskadi or basque or bilbao or bilbo or donosti* or "san sebastian" or vizcaya or bizkaia or guipuzcoa or gipuzkoa or alava or araba or vitoria or gasteiz or navarr* or nafarroa or pamplona or iruna or irunea or logron* or rioj* or aragon* or zaragoza or teruel or huesca or mancha or "ciudad real" or albacete or cuenca or (toledo not (ohio or us or usa or OH)) or (guadalajara not mexic*) or balear* or mallorca or menorca or ibiza or eivissa or palmas or lanzarote or canari* or tenerif* or ceuta or melilla) Editions: WOS.SCI,WOS.SSCI | 287.057 |
| 4 | #3 AND #2 AND #1 Editions: WOS.SCI,WOS.SSCI | 152 |
| TOTAL | | 396 |

Supplementary Table 2. Script used by the interviewer in its original language (Spanish).

| El objetivo de la reunión es ver cómo se debería preguntar por el consumo de drogas ilegales para disponer de indicadores y saber qué es lo que significan.  Hemos revisado diferentes estudios que preguntan a población española sobre el consumo de heroína y lo que vemos es que las preguntas son diferentes y no tenemos claro cómo sería mejor abordar este tema. | | |
| --- | --- | --- |
| **OBJETIVOS** | **Preguntas Generales** | **Verificación** |
|  | Los estudios diferencian momentos temporales en las encuestas de salud preguntando por el consumo alguna vez en la vida, en los últimos 12 meses, en los últimos 30 días o por el consumo actual. ¿Tenemos que preguntar por las exposiciones en el **pasado** **(seguir punto 1)** o solo en el **presente (pasar a punto 2)**? |  |
| 1. **PASADO** | PRIMER CONSUMO:   - ¿Crees que se puede recordar el momento o la edad del primer consumo? Piensa que recordar cosas que han pasado hace tiempo puede no ser fácil. - ¿Sería interesante incluir a qué momentos se asocia (momentos positivos o negativos)? Por ejemplo, celebraciones, momentos de estrés…   ETAPAS ESPECIFICAS:   - ¿Sería útil preguntar sobre el consumo durante la adolescencia? ¿Otros momentos temporales? PRENATAL, EMBARAZO. - ¿Es interesante preguntar por momentos de ocio como fiestas, vacaciones u otros momentos específicos de la vida?   TIPOS Y VÍAS:   - ¿Y por las vías de administración (inyectada, fumada, esnifada) o la cantidad administrada habitualmente cada vez? - ¿Las diferentes vías de administración a lo largo del tiempo? - ¿La cantidad administrada habitualmente cada vez? - ¿Las diferentes vías de administración a lo largo del tiempo?   CONVIVIENTES:   - Es relevante preguntar si eran consumidores:   - los padres   - otros convivientes   - las parejas   - círculo de amigos | Preguntar por el pasado: tiene sentido para poder reconstruir cómo son las historias de consumo y poder incluir medidas de prevención  Momentos temporales:   - valorar la exposición a lo largo de la vida considerando la posible presencia de sesgos de recuerdo   - Viabilidad de mitigarlo usando momentos concretos   Si empiezan por una vía y después se avanza a otras más graves... |
| 2**.PRESENTE** | DEFINICIÓN:   - Para ti , ¿qué es el presente o la actualidad? ¿Es hoy o es un día normal de “rutina”? ¿Es la semana previa? ¿El mes previo?   Nota: Generalmente las encuestas preguntan por los últimos 30 días para aproximar el presente. *“Durante los últimos 30 días, ¿ha consumido usted heroína? Sí /No”*   - En un MES se podrían incluir otros periodos de referencia: ¿últimos 7 días? ¿últimas tres semanas? - En los estudios que valoran el consumo en el último mes se pregunta por los días de consumo. ¿Esto es fácil de recordar?   ACTUAL CONSUMO:   - ¿Sería interesante incluir a qué momentos se asocia (momentos positivos o negativos)? Por ejemplo, celebraciones, momentos de estrés…   TIPOS Y VÍAS:   - ¿Y por las vías de administración (inyectada, fumada, esnifada) o la cantidad administrada habitualmente cada vez? - ¿Las diferentes vías de administración a lo largo del tiempo? - o - ¿La cantidad administrada habitualmente cada vez? - ¿Las diferentes vías de administración a lo largo del último mes? Si empiezan por una vía y después se avanza a otras más graves...   CONVIVIENTES:   - ¿Es relevante preguntar si son consumidores:   - los padres?   - las parejas?   - el círculo de amigos?   OTROS   - ¿Piensas que el consumo se oculta? - ¿Crees que sería interesante preguntar por algún factor o característica más del consumo de heroína como el tiempo máximo de abstinencia, recaídas, intentos de abandono de consumo, tratamientos previos u otros? | ¿Por qué no ve claro preguntar por el pasado?  Preguntar por el pasado tiene sentido para poder reconstruir cómo son las historias de consumo y poder incluir medidas de prevención.  Periodo de referencia: ¿Cuál es el período de referencia más idóneo para la medir el consumo presente de heroína?  Momentos temporales:  Viabilidad de mitigar sesgos de recuerdo usando momentos concretos |

Supplementary Table 3. Characteristics of the included studies (n=29).

| **First author's last name**  **(Year of publication)** | **Study design**  **(Period of execution)**  **Name of study** | **Geographical scope** | **Study population (sample size)**  **(Sex / age range or mean age in years)** | **Main objective** | **Questionnaire administration**  **(Validation)** | **Source of recruitment** | **Incen-tives** |
| --- | --- | --- | --- | --- | --- | --- | --- |
| Bravo Portela (1996) (Bravo Portela et al., 1996) | Cross-sectional (1993) | Local | Drug users (441)  (Both / mean: 27.9) | Risk practices | Face to face | Distribution and consumption settings | No |
| Royo-Bordonada (1997) (Royo-Bordonada, 1997) | Cross-sectional (1989) | National | General population (2495)  (Both / >18) | Psychoactive substance use | Face to face** | Respondents’ home | No |
| Mendoza Berjano (1998) (Mendoza Berjano et al., 1998) | Cross-sectional (1994) | National | High school students (6711)  (Both / 11, 13, 15, 17, 18) | Psychoactive substance use | Self-administered** | Educational | No |
| Moncada Ribera (1998) (Moncada Ribera & Perez Gonzalez, 1998) | Cross-sectional (1994-1995) | Local | High school students (1269)  (Both / 14-19) | Psychoactive substance use | Self-administered** | Educational | No |
| Pérez González (1999) (Perez Gonzalez et al., 1999) | Cross-sectional (1992) | Local | Drug users (NS)  (Both / mean: 27.4) | Psychoactive substance use | Face to face** | Social-health care | No |
| Bravo Portela (2000) (Bravo Portela et al., 2000) | Cross-sectional (1992-1994 and 1996) | Local | Drug users (2675)  (Both / mean: 26.8-29.5) | Risk practices | Face to face | Distribution and consumption settings | No |
| Suelves (2003) (Suelves et al., 2003) | Cross-sectional (1995, 1997, 1999)  EDD | Local | General population (NS)  (Both / 15-29) | Health Plan evaluation | Respondents’ home | Home | No |
| Bravo (2004) (Bravo et al., 2004) | Cross-sectional (1998-2000) | Regional | Drug users (1638)  (Both / mean: Galicia: 30.8; Madrid: 32.1, Sevilla: 33.3; Valencia: 32.0) | Risk practices | Face to face | Distribution and consumption settings | No |
| Prinzleve (2004) (Prinzleve et al., 2004) | Cross-sectional (NS) | Local | Drug users (1855)  (Both / 16-62) | Psychoactive substance use | Face to face | Social-health care | Yes |
|  |  |  |  |  | NS |  |  |
| Busquets (2005) (Busquets et al., 2005) | Cohortes (NS) | Local | People with mental problems (30)  (Both / >18) | Substance use and mental health problems | Face to face (Urine analysis) | Social-health care | No |
| Folch (2006) (Folch et al., 2006) | Cross-sectional (2004) | Regional | Drug users (300)*  (Both / >18) | Risk practices | Face to face** | Distribution and consumption settings | Yes |
| Garcia-Algar (2009) (Garcia-Algar et al., 2009) | Cross-sectional (2002-2004) | Local | Mothers (1209)  (Women / NS) | Psychoactive substance use | Face to face (Meconium analysis) | Social-health care | No |
| Vázquez (2010) (Vazquez, 2010) | Cross-sectional (NC) | Local | University students (559)  (Both / >18) | Psychoactive substance use | Face to face** | Educational | No |
| Etcheverry (2011) (Etcheverry et al., 2011) | Cross-sectional (2005) | Local | Drug users (326)  (Both / >18) | Psychoactive substance use | Face to face** | Social-health care | No |
| Caravaca-Sánchez (2016) (Caravaca-Sánchez & Wolff, 2016) | Cross-sectional (2014) | Regional | Inmates (2709)  (Both / >18) | Sexual violence | Self-administered** | Penitentiaries | No |
| Maremmani (2016) (Maremmani et al., 2016) | Cross-sectional (NS) | International | Drug users (126)  (Both / 18-69) | Psychoactive substance use | Self-administered | Social-health care | No |
| Caravaca-Sánchez (2018) (Caravaca-Sánchez et al., 2018) | Cross-sectional (2014) | Regional | Inmates (225)  (Women / 19-70) | Psychoactive substance use | Self-administered** | Penitentiaries | No |
| Font-Mayolas (2019) (Font-Mayolas et al., 2019) | Cross-sectional (2014-2015) | Local | University students (968)  (Both / 18-38) | Psychoactive substance use | Self-administered | Educational | No |
| Fuster-RuizdeApocada (2019) (Fuster-RuizdeApodaca et al., 2019) | Cross-sectional (2016-2017) | Regional | People with HIV(1401)  (Both / >18) | Psychoactive substance use | Self-administered | Social-health care | Yes |
| Vázquez (2019) (Vazquez et al., 2019) | Cross-sectional (2011-2012) | Local | People in unhoused situation (296)  (Both / >18) | Vulnerability | Face to face | Social-health care | No |
| Caravaca-Sánchez (2020) (Caravaca-Sanchez & Garcia-Jarillo, 2020) | Cross-sectional (2017) | Local | Inmates (174)*  (Women / mean: 37.6) | Perceived social support | Self-administered | Penitentiaries | No |
| Caravaca-Sánchez (2020) (Caravaca-Sánchez & Wolff, 2020) | Cross-sectional (2017) | Regional | Inmates (943)  (Men / 19-83) | Substance use and mental health problems | Self-administered | Penitentiaries | No |
| Guillen (2020) (Guillen et al., 2020) | Longitudinal (NS) | Local | People in unhoused situation (138)  (Women / mean: 45) | Psychoactive substance use | Face to face | Social-health care | No |
| Caravaca-Sánchez (2021) (Caravaca-Sánchez et al., 2021) | Cross-sectional (2017) | Regional | Inmates (174)  (Women / 19-58) | Substance use and mental health problems | Self-administered | Penitentiaries | No |
| Whitlock (2021) (Whitlock et al., 2021) | Cross-sectional (2018-2019)  Chem4EU | International | People with HIV (491)  (Men / >18) | Psychoactive substance use | Self-administered | Social-health care | No |
| Incera-Fernandez (2022) (Incera-Fernandez et al., 2022) | Cross-sectional (2021) | National | Drug users (1181)*  (Both / 18-78) | Risk practices | Self-administered | Internet | No |
| Parro-Torres (2022) (Parro-Torres et al., 2022) | Cross-sectional (2013-2015) | Local | People with HIV (125)  (Both / mean: 47,78) | Substance use and mental health problems | Face to face | Social-health care | No |
| Garcia-Perez (2023) (Garcia-Perez et al., 2023) | Cross-sectional (2018)  ESTUDES | National | High school students (36984)  (Both / 14-18) | Psychoactive substance use | Self-administered | Educational | No |
| Perez (2023) (Perez et al., 2023) | Cross-sectional (2022) | Local | University students (6798)  (Both / mean: 21) | Substance use and mental health problems | Self-administered | Educational | No |
| NS: not specified; EDD: Household Survey on Drug Use (*Encuesta Domiciliaria sobre Consumo de Drogas*); ESTUDES: Survey on Drug Use in Secondary Education in Spain (*Encuesta Sobre Uso de Drogas en Enseñanzas Secundarias en España*).  *Convenience sampling.  **Trained interviewer present. | | | | | | | |

Supplementary Figure 1. Number of studies published each year.
